# Rheological Properties and Antioxidant Activity of Gelatin-Based Edible Coating Incorporating Tomato (*Solanum lycopersicum* L.) Extract

**DOI:** 10.3390/gels10100624

**Published:** 2024-09-28

**Authors:** Danya E. Estrella-Osuna, Saul Ruiz-Cruz, Francisco Rodríguez-Félix, Cielo E. Figueroa-Enríquez, Humberto González-Ríos, Daniel Fernández-Quiroz, Enrique Márquez-Ríos, José Agustín Tapia-Hernández, José Ángel Pérez-Álvarez, Guadalupe Miroslava Suárez-Jiménez

**Affiliations:** 1 Department of Research and Graduate Studies in Food, University of Sonora, Encinas y Rosales s/n, Hermosillo 83000, Sonora, Mexico; a222230197@unison.mx (D.E.E.-O.); a214200337@unison.mx (C.E.F.-E.); enrique.marquez@unison.mx (E.M.-R.); joseagustin.tapia@unison.mx (J.A.T.-H.); miroslava.suarez@unison.mx (G.M.S.-J.); 2 Centro de Investigación en Alimentación y Desarrollo, A.C, Carretera Gustavo Enrique Astiazarán Rosas 46, Hermosillo 83304, Sonora, Mexico; hugory@ciad.mx; 3 Department of Chemical Engineering and Metallurgy, University of Sonora, Av. Colosio S/N, Centro, Hermosillo 83000, Sonora, Mexico; daniel.fernandez@unison.mx; 4 IPOA Research Group, Grupo 1-UMH Grupo REVIV, Generalitat Valenciana, Dpto Tecnología Agroalimentaria, Escuela Politécnica Superior de Orihuela, Universidad Miguel Hernández, Ctra Beniel, Km 3.2, E-03312 Orihuela, Alicante, Spain; ja.perez@umh.es

**Keywords:** biopolymer, bioactive compounds, coatings, viscosity, antioxidants, gels

## Abstract

Gelatin is a promising biopolymer for edible coatings thanks to its low cost and gelling properties. However, its weak mechanical properties limit its use. This study aimed to develop a gelatin coating with tomato extract, analyzing its antioxidant activity and rheological properties for food applications. Gelatin concentrations (2, 5, and 7%) were evaluated, and it was determined that 7% with 7.5% glycerol was the optimal mixture. Three concentrations of tomato extract (0.5, 1, and 1.5%) were added, and antioxidant activity was evaluated using the ABTS technique, as well as the interaction of components through FT-IR and physicochemical analysis. The results showed that there were no significant differences in terms of their physicochemical characterization, maintaining a pH of 5 and a yellowish hue. The FT-IR spectra indicated there were hydrogen bond interactions between gelatin and the extract. The antioxidant capacity was higher with the 1.5% extract, achieving an inhibition of 58.9%. It was found that the combination of the different materials used improved the rheological (specifically the viscosity and stability of the material) and antioxidant properties of the gelatin. These findings suggest that modified gelatin coatings may be effective in extending the shelf life of foods.

## 1. Introduction

All food products are perishable and susceptible to rapid deterioration in quality, so the packaging that preserves them decides the active preservation of their properties [1]. In recent years, research on active coatings or packaging has presented its high potential to delay the deterioration of food, thus prolonging its useful life [2]. These coatings can be defined as thin layers of edible materials with a thickness generally less than 0.3 mm that can alter the molecular exchange between food and the environment and its behavior, aiding in freshness, transportation, and storage. More specifically, they reduce or inhibit oxygen, lipids, aromas, and moisture migration since they create a semipermeable barrier. Likewise, they can act as a vehicle for active agents such as antimicrobials, pigments, and antioxidants [3,4]. These edible coatings can be prepared from biopolymers, which are usually generated from food industry waste, or sources of proteins, lipids, or polysaccharides [5]. Gelatin is one of the most used ingredients in edible coatings applied to fresh products. It is a water-soluble, colorless, and tasteless protein produced from the partial hydrolysis of collagen [6]. It is a biodegradable polymer that is inexpensive to produce, highly available, and easy to access.

Gelatin is divided into two types according to its production process: type A and B. Type A is obtained from an acid extraction, with an isoelectric point between 6 and 9. It is obtained from covalently linked collagen with a lower degree of cross-linking, generally being made of pigskin. Type B gelatin is obtained from an alkaline extraction, with an isoelectric point of pH 5. It is obtained from a more complex collagen, generally present in cow skin. Therefore, type B gelatin has a better variety of physical and chemical, film-forming, emulsion, and foaming properties, and it acts as a gas barrier [4,7]. However, it suffers from weak mechanical strength and water vapor permeability, so its use as a packaging material may be limited, but it can be improved by mixing gelatin with other functional materials and active ingredients.

Adding natural extracts can improve the functional and physical properties of edible coatings, especially if they are rich in bioactive compounds [8]. It has been reported that tomatoes have a significant content of active compounds such as antioxidants, more specifically phenolic compounds (chlorogenic, coumaric, and caffeic acid), vitamins C and E, flavonoids, fiber, and protein [9]. They also contain carotenoids, especially lycopene (a compound that represents approximately 90% of the carotenoids present in tomatoes) [10]. Therefore, tomato extracts could be a good alternative for use in edible coatings. It has been reported in recent years that the extraction of these types of compounds has been used for the design and fortification of foods. Some studies mention that the incorporation of carotenoids present in tomatoes in films and/or edible coatings improves their mechanical properties and coloration and acts as a barrier against light [11]. Gallego et al. [12], reported an example of this involving the development of a gelatin coating enriched with antioxidant by-products of tomato applied to a pork model. The application of the coating showed physicochemical changes such as greater weight loss, but the pH and water activity were maintained. Likewise, the coating prevented lipid oxidation and contributed a higher antioxidant activity. Canché-López et al. [13] developed edible films based on chitosan incorporating natural extracts of tomato and moringa. When applying this coating to pork loin, they observed a decrease in water loss and a decrease in microbial growth in the coated samples. It was also observed that at higher concentrations of glycerol, the samples presented good material properties, which made the coating suitable for application.

For this reason, rheological study has been positioned in one of the crucial stages in the development of materials since it allows us to perceive the relationship between the structure (viscous and elastic properties) and function (thickness and uniformity) of a solution [14].

Therefore, this work aimed to develop an edible coating based on gelatin combined with tomato extract to characterize it in terms of its antioxidant activity and rheological properties to predict its potential applications within the food industry.

## 2. Results and Discussion

### 2.1. Rheology of Coating Solutions

**Figure 1:**
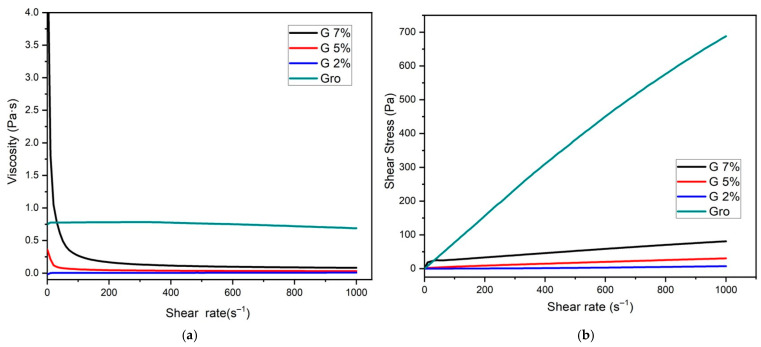
Rheogram of gelatin (G) at different concentrations (2, 5, and 7%) and glycerol (Gro), where (**a**) is the flow viscosity vs. the shear rate, and (**b**) is the shear stress vs. the shear rate.

Figure 1 shows the viscosity curves as a function of the shear speed applied at a temperature of 25 °C. As expected for polymer solutions, in this case, gelatin, shear thinning was observed, which means a decrease in viscosity according to the increase in shear rate. It has been reported that an increase in cutting speed leads to greater ordering of the polymer chains, which tend to orient themselves towards the applied tension. The greater their degree of orientation, the lower their viscosity [15]. Furthermore, the alignment of large-molecule chains at a higher shear rate generates higher resistance, and, therefore, the material’s viscosity decreases [16]. Furthermore, the observed behavior can be corroborated by applying the power law, which describes the variation in the viscosity of a non-Newtonian fluid with the deformation rate (Table 1). According to the applied power law, the one with the highest value was glycerol, followed by the selected gelatin concentrations. Glycerol presented a Newtonian behavior, which is characteristic of this type of material since its viscosity always remains constant. On the other hand, each gelatin concentration had a value less than 1, which indicates that a material is a pseudoplastic fluid, a state that depends on the value of the fluid according to the concentration used. Specifically, gelatin, in its use for edible coatings, could vary between having a K (consistency constant) between 1 and 10 Pass^n^, which depends on the formulation used and the concentration. The value n (the flow exponent) generally ranges between 0.5 and 1.0. A value of n less that is than 1 indicates pseudoplastic behavior, meaning that the viscosity decreases with increasing shear stress, while a value of n = 1 indicates that it behaves like a Newtonian fluid, meaning that its viscosity is constant and does not change with shear stress [17].

**Figure 2:**
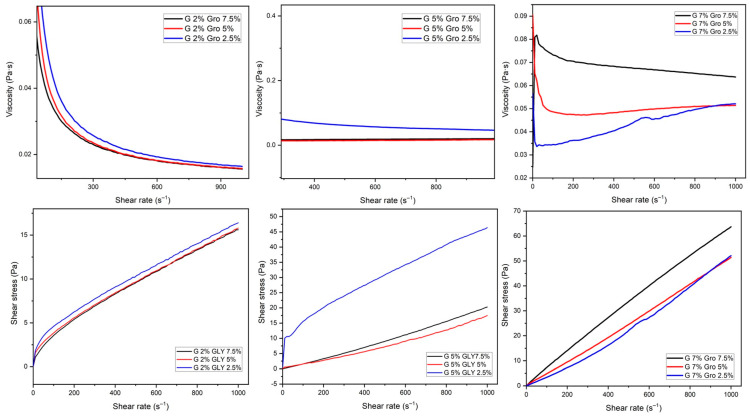
Rheogram of solutions prepared from gelatin (G) (2, 5, and 7%) and glycerol (Gro) (2.5, 5, and 7.5%).

Figure 2 shows the interaction of the nine formulations made of gelatin (2, 5, and 7%) and incorporating glycerol (2.5, 5, and 7.5%). Similar pseudoplastic behaviors can be observed (Table 2) for the formulations made of 2 and 5% gelatin using glycerol at 2.5 and 5% since their viscosity was decreased when applying the shear force. The opposite was observed in the combination of 7% gelatin with the three glycerol concentrations, yielding a solution with a more stable viscosity according to Newton’s law regarding a Newtonian fluid. And this may be due to the concentration of gelatin since it is known that a higher concentration results in a higher viscosity, which is dependent on the concentration, temperature, and Bloom’s value [16]. In the same sense, plasticizers reduce the intermolecular forces established between the polypeptide chains of gelatin, generating greater mobility, which improves flexibility and helps maintain integrity and produce more-uniform coatings [18]. Glycerol is highly effective as a plasticizer and has a good affinity for protein matrices, with the ability to interact specifically with hydrogen bonds [19].

**Figure 3:**
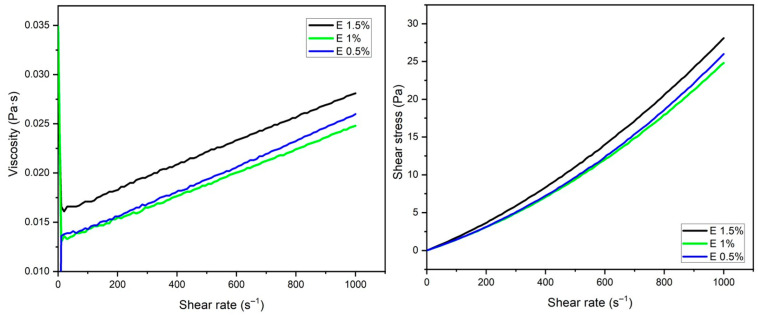
Rheogram of solutions prepared from 7% gelatin and 7.5% glycerol supplemented with extract (0.5, 1.0, and 1.5%).

For the addition of the tomato extract, the most stable formulations were selected to maintain a better viscosity, namely, gelatin at 7% and glycerol at 7.5%, to which extract concentrations of 0.5, 1, and 1.5% were added. A significant improvement in viscosity stability was observed (Figure 3), presenting larger regions of Newtonian fluids for the three concentrations (Table 3). It has been reported that the addition of natural extracts can improve the physical and functional properties of edible coatings. They improve adhesion by acting as binding agents. Also, depending on the extract used, they can contribute to improving the texture and viscosity of a coating. Also, thermal stability and strength are influenced, helping to prevent breakage or disintegration during storage and handling [8]. In the same sense, the addition of an extract has a plasticizing effect that reduces interchain forces by interrupting the bonds, reducing the cohesive strength of the polymer matrix and, therefore, its resistance to fracturing [20]. High-viscosity coatings can improve performance by reducing friction, increasing heat transfer, and providing better adhesion with other materials.

### 2.2. Interaction of Added Coating Solution with Tomato Extract

**Figure 4:**
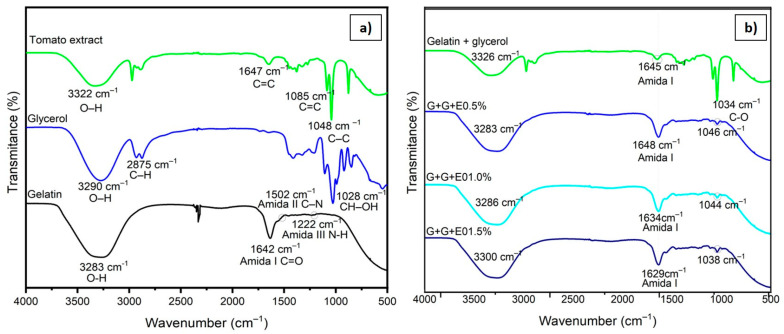
(**a**) Infrared spectra of gelatin, glycerol, and tomato extract and (**b**) gelatin and glycerol emulsion (7 and 7.5) with tomato extract (0.5, 1.0, and 1.5%).

In this analysis, infrared spectroscopy was employed to identify key functional groups by correlating transmittance percentages with specific wavelengths (expressed in cm^−1^). The absorption bands observed in the IR spectra represent the characteristic vibrations of the atoms within the samples’ molecular structures. These spectra are displayed in Figure 4a, showing the materials used, and Figure 4b, which includes the spectra after the addition of three different concentrations of tomato extract. First, focusing on the tomato extract, a prominent absorption band is observable at 3322 cm^−1^. This band is indicative of hydroxyl (OH) groups, which are likely present due to the ethanol used as an extraction solvent. Ethanol contains hydroxyl functional groups that could contribute to this specific absorption. The peak at 1647 cm^−1^ corresponds to the stretching vibrations of carbon–carbon double bonds (C=C), typical in organic molecules with conjugated systems. Additionally, the band at 1085 cm^−1^ is associated with the stretching vibrations of an aromatic ring structure, which may arise from phenolic or other aromatic compounds naturally present in the extract. The key absorption band at 1048 cm^−1^ can be attributed to the C–C stretching in the lycopene rings. Lycopene, a major carotenoid in tomatoes, contains a series of conjugated double bonds, and its molecular structure contributes to these distinctive IR signatures.

Gelatin, being a protein, exhibits characteristic bands typically associated with its peptide bonds. The amide I band, which results from the stretching of carbonyl groups (C=O), was observed at 1642 cm^−1^. This band is a hallmark of proteins and reflects the backbone structure of the polypeptide chain. The amide II band, occurring at 1502 cm^−1^, is present due to N–H bending and C–N stretching vibrations. Meanwhile, the amide III band, observed at 1222 cm^−1^, corresponds to a combination of N–H bending and C-N stretching. This detailed profile provides insight into the molecular vibrations associated with protein structures. In addition, the band at 3283 cm^−1^ reflects both –OH and –NH groups, likely due to vibrational stretching, further characterizing gelatin’s polar functional groups. Upon examining the union of gelatin with glycerol, a noticeable increase in the intensity of the band at 3326 cm^−1^ can be observed, signifying enhanced NH stretching, likely resulting from the formation of hydrogen bonds between the gelatin and glycerol. Glycerol’s hydroxyl groups are highly polar and capable of forming strong hydrogen bonds with the amide groups of gelatins, leading to the observed increase. Additionally, an intensification of the band at 1034 cm^−1^ is evident, which can be attributed to the interaction between the carbonyl (CO) stretching in glycerol and the gelatin structure. This interaction indicates that glycerol, acting as a plasticizer, was integrated into the gelatin matrix, altering its molecular vibrations.

In coatings that incorporate tomato extract, the presence of lycopene is confirmed by characteristic absorption bands between 1200 cm^−1^ and 700 cm^−1^, particularly around 1040 cm^−1^. These bands are associated with the molecular structure of lycopene, specifically its conjugated double bonds, which are responsible for this compound’s optical and chemical properties. The observation of these spectral signals indicates the successful encapsulation or entrapment of lycopene within the gelatin–glycerol matrix, suggesting that this bioactive compound was effectively integrated into the polymeric system. Moreover, the incorporation of tomato extract not only introduces lycopene into the matrix but also causes subtle shifts in the bands traditionally attributed to gelatin. These spectral shifts may be related to conformational changes in the protein’s structure, implying an interaction between the components of the tomato extract and the gelatin. Specifically, interactions between the functional groups of lycopene, as well as other potential compounds present in the extract, such as flavonoids or organic acids, could alter the network of hydrogen bonds and intermolecular interactions within the gelatin. This alteration in the protein’s structural arrangement could influence its mechanical and physical behavior, as well as affect the distribution of glycerol, which acts as a plasticizer in the mixture. Consequently, the modification of the gelatin structure may impact key properties of the coating, such as its flexibility, strength, and permeability, suggesting a direct relationship between chemical interactions and the final performance of the material.

In discussing the incorporation of natural extracts into gelatin matrices, it is important to highlight several recent studies that have shown similar results regarding spectral interactions, the types of interactions observed, and functional properties. For instance, Hamann et al. [2] developed edible films based on green tea extract and gelatin for coating fresh sausages. FTIR analysis revealed shifts in the bands associated with gelatin, indicating there were hydrogen bonding interactions between the bioactive compounds in the tea and the protein matrix. These changes align with the findings of this study, where lycopene from tomato extract induced similar hydrogen bonding modifications. Additionally, Nunes et al. [21] investigated the effect of green tea extract on gelatin films enriched with lemon essential oil. Their results demonstrated spectral shifts, suggesting that interactions such as van der Waals forces and hydrogen bonds between the extract components and gelatin altered the film’s structure. This is comparable to the observations made regarding tomato extract in this work, where similar interactions influenced the gelatin matrix. Moreover, Zhong et al. [22] characterized gelatin films supplemented with protocatechuic acid, reporting antioxidant and antibacterial properties. Their FTIR analysis revealed changes in spectral bands that evidenced hydrogen bonding and possibly ionic interactions between the acid and gelatin. This reinforces the idea that natural extracts can modify the structural network of gelatin, which is consistent with the observations in this study regarding the effect of lycopene. Finally, Chou et al. [23] analyzed fish-skin-gelatin- and guava-leaf-extract-based bio-composite films incorporating catechin. Their results indicated changes in the FTIR bands, reflecting interactions such as hydrogen bonds and π-π stacking between catechin and the gelatin matrix. These findings align with the results observed in this work, where interactions of lycopene from the tomato extract also led to modifications in the gelatin structure, suggesting that the addition of bioactive compounds can significantly influence the mechanical and functional properties of coatings.

### 2.3. Zeta Potential

**Figure 5:**
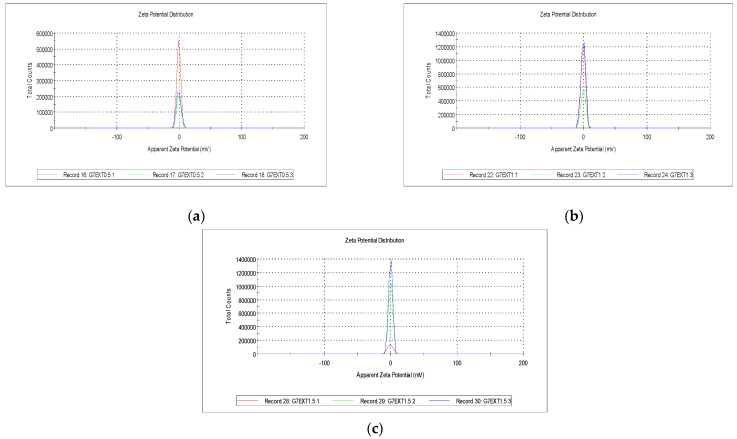
(**a**) Zeta potentials of the gelatin coating with 0.5% tomato extract, (**b**) 1% tomato extract, and (**c**) 1.5% tomato extract.

Figure 5 shows the determination of the Z potential in the gelatin-based coatings supplemented with three concentrations of extract (0.5, 1, and 1.5%), revealing results with a significant difference, with z potential values of −0.757 ± 0.0445, −0.321 ± 0.0807, and −0.0785 ± 0.0531, respectively, which highlight the complexity of the interactions in the three systems, suggesting a possible correlation between surface charge and extract concentration. Therefore, there are notable differences between the samples, suggesting variations in the gelatin suspensions’ colloidal stability with respect to the extract. These findings indicate a possible influence of extract concentration on the surface charge of the gelatin particles, which, in turn, could affect the electrostatic repulsion and stability of the suspension at higher extract concentrations [24].

Furthermore, the adhesion and cohesion of the gelatin coating are expected to vary with extract concentration due to changes in the structure and distribution of the particles when incorporated into the surface of a substrate. These results contribute to what was previously reported in the literature on gelatin coatings, highlighting the importance of understanding how the incorporation of an extract affects not only the physical properties of a coating, such as viscosity, apparent density, and water retention capacity, but also its stability and interfacial behavior. Previous research, such as the study by Pérez-Marroquín et al. [25], also found a reduction in the z-potential of colloidal particles when modifying the coatings with natural extracts. In gelatin-based coatings, the optimal z-potential value is generally slightly away from zero, favoring the colloidal suspensions’ stability and the coating’s adhesion to the substrate. When the z-potential is far from zero, the gelatin particles in the suspension will have similar electrical charges, resulting in the generation of electrostatic repulsion forces between them. These forces help prevent particle accumulation and settling, contributing to better suspension stability and uniform coating distribution over a substrate’s surface.

### 2.4. Antioxidant Capacity

The content of total phenolic compounds in the tomato extract was 114.15 mg EAG/100 gfw. Dominguez et al. [26] mentioned that the main compounds present in tomatoes are hydroxycinnamic, chlorogenic, p-coumaric, ferulic, and cafeic acids, occurring in higher concentrations in peels and seeds. Similar values were reported in the study by Katirci et al. [9], with an ethanolic extract with 112 mg of EAG/100 gfw; the opposite occurred in what was reported by Rojas et al. [27], with 42.74 mg of EAG/100 gfw, attributing this behavior to the extraction solvents as well as the applied mechanical work. In the same sense, the variety of tomato employed can significantly affect the content of these compounds.

On the other hand, when carrying out the radical inhibition assay of the extract, 82.54 and 51.29% inhibition were obtained, corresponding to DPPH and ABTS, respectively. It is known that antioxidant activity is closely related to phenolic compounds. Likewise, combining compounds such as lycopene, carotenoids, and vitamins present in tomato extract can enhance its antioxidant effects [28]. Therefore, the phenolic compounds in tomato extract exert antioxidant activity. These compounds can act by interrupting oxidation chain reactions as a hydrogen atom donor, an acceptor of free radicals, or by chelating metals [29]. Considering this phenolic content and the antioxidant capacity of tomato extract, the reaction of the union of these compounds with the preparation of the polymeric matrix of the edible coating, gelatin, was analyzed. Only an ABTS analysis was carried out to determine the antioxidant activity of the selected formulation since the DPPH analysis showed interference, as gelatin is a non-soluble protein in methanol solutions. Furthermore, for measuring total antioxidant capacity, the ABTS method is more efficient in terms of hydrophilic and lipophilic antioxidants. It has already been reported that gelatin alone exhibits antioxidant activity, reporting in this study an inhibition value of the ABTS radical of 52.06%, which increased when adding the three concentrations of extract (0.5, 1, and 1.5%), rising to 53.87, 55.87, and 58.91% respectively. The amino acids present in gelatin protein can provide coatings and/or films with poor antioxidant activity, which can be improved when they are conjugated with various phenolic compounds. A study carried out by Hanani et al. [30] evaluated the antioxidant capacity of gelatin-based films supplemented with pomegranate powder, where only the control (gelatin-only) showed 32% inhibition of the ABTS radical, and this result increased as they incorporated pomegranate powder, achieving a maximum value of 80% inhibition. This is the same behavior observed in this study.

### 2.5. Physicochemical Characterization

The physicochemical characterization of the materials and formulations made of gelatin, glycerol, and tomato extract at different concentrations (0.5, 1.0, and 1.5%) can be seen in Table 4. Regarding the color of an edible coating, something important to mention is that the coatings must be a light color to avoid altering the color of food. There were no significant differences based on the added concentration of tomato extract; however, there was a slight increase in gelatin alone. Likewise, it can be seen that as the extract concentration increased, there was a reduction in luminosity (L*). Therefore, the coating showed a yellowish hue. The color, gloss, and transparency of edible coatings vary significantly depending on the chemical composition and structure of the polymer used [31]. Regarding color behavior, similar studies have mentioned that the presence of yellowish hues is due to the presence of lycopene and astaxanthin [32]. Likewise, there was no significant difference in the pH, and it was observed that the manufacturing material and the formulations had a pH of around 5. Something significant to consider is that depending on the pH of the solution from which it is created, a coating’s properties, such as its color and texture, can change [33].

## 3. Conclusions

Gelatin is a very promising biopolymer for creating edible coatings due to its low cost, polymerization ability, and inherent antioxidant properties. However, its application is often hindered by its weak mechanical properties. This study demonstrated that adding active ingredients, such as glycerol and tomato extract, can significantly enhance gelatin’s stability and antioxidant capacity. Specifically, a 7% gelatin concentration mixed with 7.5% glycerol proved to be a stable Newtonian fluid, and incorporating 1.5% tomato extract led to the highest antioxidant activity. These findings suggest that modifying gelatin-based coatings with natural extracts could be effective in extending the shelf lives of foods by maintaining desirable physicochemical properties and enhancing antioxidant effects.

## 4. Materials and Methods

### 4.1. Obtaining Materials and Reagents

The tomatoes were obtained from a local supermarket in Hermosillo, Sonora, Mexico, at an overripe maturity stage. Gelatin type B; glycerol; 2, 2-diphenyl-1-picrylhydrazyl (DPPH); 2, 2-azino-bis (3-ethylbenzothiazoline-6-sulfonic acid) (ABTS); and the Folin–Ciocalteu reagent were obtained from Sigma-Aldrich (St. Louis, MO, USA). Ethanol (99% *v*/*v*) and ethyl acetate (99% *v*/*v*) were procured from Mayer (México City, México). Distilled water was also used.

### 4.2. Ultrasound-Assisted Preparation of Tomato Extract

**Figure 6:**
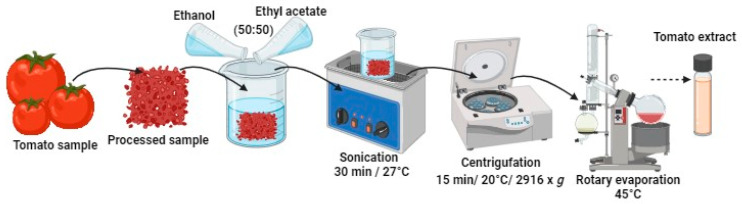
Illustration of the extraction process of active compounds from tomato.

Following the methodology employed by Silva et al. [10], the tomato samples were crushed until constituting a homogeneous paste. Ethyl acetate and ethanol (99%) were used as an extraction solvent. The mixing of solvents was carried out before their use in a ratio of 1:1 (*v*/*v*). A total of 10 g of processed tomato were placed, and 100 mL of the solvent mixture was added and stirred manually for 10 s. The mixture was then placed in an ultrasonic (Generator ultrasonic pulses Branson Digital Sonifier Qsonica, LLC., Danbury, CT, USA) bath at 27 °C for 30 min. At the end of the extraction time, it was centrifuged at 2916× *g* for 15 min at 25 °C, separating the sediment from the extract. Rotavaporation was used to remove the solvent, and it was refrigerated until use (Figure 6).

### 4.3. Preparation of the Edible Coating

**Figure 7:**
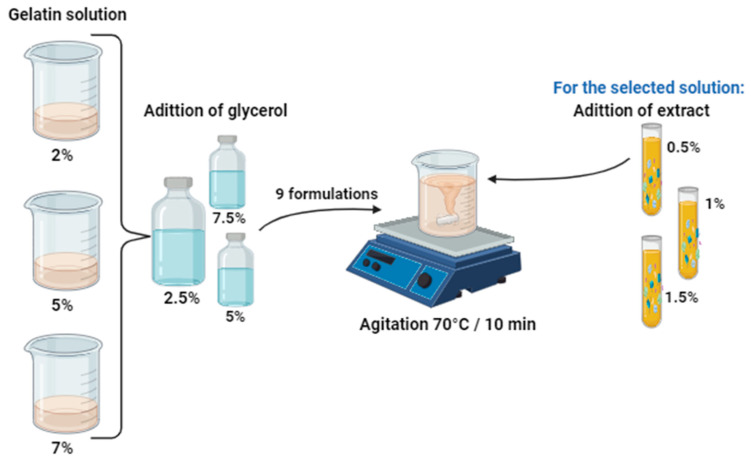
Graphic representation of the coating preparation process with different concentrations of gelatin and glycerol and, for the selected formulation, the addition of extract.

To make the edible coating, 9 formulations were prepared, for which the concentrations of gelatin (2–5% *w*/*v*) and glycerol (2.5–7% *w*/*v* gelatin) were varied. The gelatin powder was dissolved in water at approximately 70 °C with constant stirring until it was completely dissolved (approximately 20 min). Subsequently, glycerol was added, and the solution was homogenized again until it became a film-forming solution, according to the methodology of Hamann et al. [2], with modifications. After defining the concentrations of gelatin and glycerol, the tomato extract was added at 0.5, 1.0, and 1.5% *v*/*v* of the film-forming solution, leaving it to be stirred for 10 min for incorporation (Figure 7).

### 4.4. Rheology of Coating Solutions

Rheological behavior was determined using an MCR-102 rheometer (Anton Paar, Germany) with concentric cylinder geometry. Shear rate versus shear stress was compared with a continuous ramp from 0.1 to 100 s^−1^ at 25 °C. The data obtained were adjusted to a Power Law model to deduce the behaviors of the solutions prepared (Newtonian or non-Newtonian). The effect of shear stress on viscosity was analyzed using a continuous ramp from 0.1 to 100 s and a constant shear rate of 50 s^−1^ at 25 °C. The rheometer was operated in controlled shear rate (CSR) mode, allowing precise control of shear stress and strain to capture the material’s flow properties. Temperature control was maintained throughout the experiment to ensure the accuracy of the rheological measurements. For each measurement, 100 points were used [34].

### 4.5. Fourier Transform Infrared Spectroscopy (FT-IR)

To observe the physical interaction between gelatin, glycerol, and tomato extract, FT-IR spectroscopy was used. A Spectrum GX FT-IR infrared spectrometer (Perkin-Elmer, Waltham, MA, USA) was used, and spectrum scans were performed in the range of 4000 to 500 cm^−1^. The method employed was attenuated total reflectance (ATR). The measurements were carried out in transmittance mode [35].

### 4.6. Zeta Potential

Zeta potentials were determined using a Zetasizer Nano ZS (Malvern Instruments Ltd., Worcestershire, UK). A sample (1.2 mg) was taken, dissolved in 2 mL of 95% ethanol, and placed in a polystyrene bucket to be read using the equipment. All results were recorded as the average of 5 measurements [36].

### 4.7. Antioxidant Activity

The total quantity of phenolic compounds was quantified using the Folin–Ciocalteu technique. A total of 10 µL of tomato extract was added to 25 µL of Folin solution. The solution was left to stand for 5 min. Subsequently, 25 µL of 20% Na_2_CO_3_ and 140 µL of distilled water were added, making a total volume of 200 µL. The reaction was left to stand for 30 min, and an absorbance reading was taken at 760 nm in a microplate reader (Thermo Fisher Scientific Inc. Multiskan GO. Waltham, MA, USA). The concentration of total phenols was quantified using a gallic acid standard curve, and the results were expressed as mg of gallic acid equivalent per g of fresh weight (mg/GAE/gpw), as Zhang et al. [37] described.

For the ABTS radical inhibition test, 19.3 mg of radical was dissolved in 5 mL of distilled water, while 0.0378 g of potassium persulfate and 1 mL of distilled water were mixed. Subsequently, 88 µL of persulfate was added to the ABTS solution, which was left to stand for 12 h. After some time, 270 µL of the radical solution was extracted, and 20 µL of the sample was added to a microplate. It was allowed to rest for 30 min, and an absorbance reading was taken at 734 nm [38].

Furthermore, a DPPH radical inhibition test was carried out, where the radical was first prepared, with 1.5 g of DPPH radical poured into 50 mL of methanol and adjusted to an absorbance of 0.7 ± 0.01 at 515 nm. Then, 200 µL of radical and 20 µL of sample were added to a microplate. The mixture was left to rest for 30 min, and absorbance was read at 515 nm [38].

### 4.8. Physicochemical Characterization of Coating Solutions

#### 4.8.1. Color

Color acquisition was determined by using a digital colorimeter (Beley, FRU, WR10QC, Guangzhou, China), taking into consideration the CIE LAB scale parameters (L* a* and b*), where the following scheme applies: L* (luminosity, white-black), a* (−a = green, +a = red), and b* (−b = blue and +b = yellow).

#### 4.8.2. pH

The pH was determined using a potentiometer (Hanna, Smithfield, RI, USA): 1 g of the sample was extracted, and 10 mL of distilled water was added to take a reading.

### 4.9. Experimental Design and Statistical Analysis

In this research, 3 repetitions were employed for each analysis described. A completely random design was applied. The statistical significance of the differences presented in each treatment were determined by using an ANOVA followed by a Tukey test, obtaining a comparison of the mean values. The results are expressed as mean values ± their standard deviations, with a significance level of <0.05.

## Figures and Tables

**Table 1:** Rheological behavior of gelatin (2, 5, and 7%) and glycerol.

Material Concentration (*w*/*v*)	K (pass^n^)	N	R^2^
Glycerol	0.8315 ± 0.001 ^a^	0.9827 ± 0.123 ^a^	0.9992 ± 0.023 ^a^
Gelatin 2%	0.0129 ± 0.011 ^d^	0.8345 ± 0.002 ^b^	0.8923 ± 0.019 ^d^
Gelatin 5%	0.2382 ± 0.042 ^c^	0.6953 ± 0.031 ^d^	0.9918 ± 0.022 ^b^
Gelatin 7%	0.5621 ± 0.029 ^b^	0.7884 ± 0.008 ^c^	0.9408 ± 0.320 ^c^

Note: The means in each column with a different superscript letter are significantly different (*p* ≤ 0.05).

**Table 2:** Rheological behavior of gelatin (2, 5, and 7%) and glycerol (2.5, 5, and 7.5%) solutions.

Formulation Gelatin/Glycerol (*v*/*v*)	K (pass^n^)	N	R^2^
2%/2.5%	0.2706 ± 0.0026 ^b^	0.6929 ± 0.0091 ^c^	0.9711 ± 0.0022 ^ab^
2%/5%	0.1836 ± 0.0816 ^c^	0.6442 ± 0.0075 ^c^	0.9735 ± 0.0148 ^ab^
2%/7.5%	0.1012 ± 0.0147 ^c^	0.6831 ± 0.0089 ^c^	0.9874 ± 0.0036 ^a^
5%/2.5%	0.0885 ± 0.0024 ^c^	0.7865 ± 0.0035 ^b^	0.9751± 0.0037 ^ab^
5%/5%	0.0643 ± 0.3212 ^cd^	0.7934 ±0.0052 ^b^	0.9259 ± 0.0336 ^b^
5%/7.5%	0.5097 ± 0.0058 ^a^	0.7755 ± 0.0078 ^b^	0.9830 ± 0.0787 ^a^
7%/2.5%	0.0297 ± 0.078 ^d^	1.0622 ± 0.9812 ^a^	0.9910 ± 0.7912 ^a^
7%/5%	0.0620 ± 0.0091 ^cd^	0.9610 ± 0.0156 ^a^	0.9974 ± 0.0339 ^a^
7%/7.5%	0.0586 ± 0.0872 ^cd^	1.0238 ± 0.0369 ^a^	0.9924 ± 0.0085 ^a^

Note: The means in each column with a different superscript letter are significantly different (*p* ≤ 0.05).

**Table 3:** Rheological behavior of solutions prepared with 7% gelatin, 7.5% glycerol, and tomato extract (0.5, 1.0, and 1.5%).

Formulation Gelatin/Glycerol/Extract (*v*/*v*)	K (pass^n^)	N	R^2^
7%/7.5%/0.5%	0.0113 ± 0.002 ^b^	1.0901 ± 0.0297 ^a^	0.9866 ± 0.0311 ^a^
7%/7.5%/1.0%	0.0126 ± 0.001 ^b^	1.0675 ± 0.0336 ^a^	0.9828 ± 0.0862 ^a^
7%/7.5%/1.5%	0.1432 ± 0.008 ^a^	1.0725 ± 0.0785 ^a^	0.9893 ± 0.0741 ^a^

Note: The different superscript letters are the significant difference between the means statistically determined.

**Table 4:** Physicochemical characterization of the materials and formulation made of gelatin; glycerol; and tomato extract (0.5, 1.0, and 11.5%).

Material and Formulation (Gelatin/Glycerol/Extract)	Color			pH
	L	a*	b*	
Gelatin 7%	37.58 ± 0.01 ^d^	0.61 ± 0.12 ^b^	2.43 ± 0.71 ^e^	5.46 ± 0.25 ^d^
Glycerol	20.56 ± 0.07 ^e^	−4.58 ± 0.32 ^d^	7.91 ± 0.32 ^b^	5.23 ± 0.21 ^e^
Tomato extract	40.62 ± 0.91 ^c^	21.76 ± 1.48 ^a^	10.0 ± 0.59 ^a^	6.32 ± 0.55 ^a^
Gelatin/Glycerol/Ext 0.5%	42.27 ±0.02 ^a^	0.27 ± 0.02 ^c^	3.56 ± 0.23 ^d^	5.65 ± 0.12 ^b^
Gelatin/Glycerol/Ext 1.0%	41.82 ± 0.03 ^b^	0.32 ± 0.36 ^c^	3.61 ± 0.25 ^d^	5.52 ± 0.15 ^c^
Gelatin/Glycerol/Ext 1.5%	40.12 ± 0.03 ^c^	0.39 ± 0.14 ^c^	3.85 ± 0.14 ^c^	5.42 ± 0.13 ^d^

Note: The different superscript letters are the significant difference between the means statistically determined.

## Data Availability

The original contributions presented in the study are included in the article, further inquiries can be directed to the corresponding author/s.
